# Proliferative Verrucous Leukoplakia: A Diagnostic Challenge in a Clinical and Histopathological Context—With Reflections on the Health Reality in Brazil

**DOI:** 10.1155/2024/9166581

**Published:** 2024-07-08

**Authors:** Michele Di Benedetto, Gabriela de Figueiredo Meira, Milena Martins da Rocha, Mariel Ruivo Biancardi, Jéssica Barroso Barbosa, Jeconias Câmara, Gerson de Oliveira Paiva Neto, Roberto Luiz de Menezes Martinho, Cássia Maria Fischer Rubira, Silvia Helena de Carvalho Sales Peres

**Affiliations:** ^1^ Department of Surgery Stomatology Pathology and Radiology Bauru School of Dentistry of São Paulo University FOB-USP, Alameda Dr. Octávio Pinheiro Brisolla, 9-75, Vila Regina, Bauru, SP 17012-230, Brazil; ^2^ Department of Pediatric Dentistry Orthodontics and Public Health Bauru School of Dentistry of São Paulo University FOB-USP, Alameda Dr. Octávio Pinheiro Brisolla, Block 9, Jardim Brasil, Bauru, SP 17012-901, Brazil; ^3^ School of Dentistry of FAMETRO Metropolitan University of Manaus FAMETRO, Av. Constantino Nery, n° 3000, Chapada, Manaus, AM 69050-000, Brazil; ^4^ Department of Pathology and Legal Medicine of Amazonas Federal University FM-UFAM, Avenida Apurinã, n° 4, Praça 14 de Janeiro, Manaus, AM 69020-170, Brazil; ^5^ School of Medicine of Amazonas Federal University UFAM-ISB, Estr. Coari/Mamiá, n° 305, Espírito Santo, Coari, AM 69460-000, Brazil

## Abstract

Proliferative verrucous leukoplakia (PVL) is an aggressive and distinct oral disorder with a high potential for malignant transformation (MT). It presents as multifocal lesions that progress over time and frequently recur, often developing carcinomas. Accurately diagnosing PVL is crucial to distinguish it from other oral mucosa lesions that have a lower risk of cancer progression. However, due to the diverse histological features observed in PVL, identifying clinical criteria and histological patterns that can be applied by unfamiliar professionals is challenging. In this study, we present a case of PVL associated with dysplasia in a 53-year-old female patient. The patient exhibited macular and leukoplakic nonscrapable lesions disseminated throughout the oral cavity, with continuous growth. The diagnosis of PVL was established during an 18-month follow-up. This case highlights the difficulties faced by both clinicians and pathologists in diagnosing PVL, emphasizing the need for careful evaluation and accurate diagnosis, particularly in patients with unusual oral lesions, and highlighting the discrepancies observed in the application of available protocols to our particular case. Distinguishing PVL from similar conditions can be challenging due to overlapping clinical signs and indistinct histopathological features.

## 1. Introduction

Proliferative verrucous leukoplakia (PVL) is a subtype of oral leukoplakia. Recognized by the World Health Organization (WHO) in 2005 and reaffirmed in a 2020 consensus meeting in Glasgow, PVL falls within the spectrum of oral potentially malignant disorders (OPMDs) [1].

PVL was first described by Hansen et al. in 1985 [2]. Since then, multiple diagnostic criteria have been suggested [3]. Currently, PVL is recognized as a distinct multifocal oral leukoplakia with a progressive clinical course, evolving clinical and histopathological traits [4]. It has a higher probability of malignant transformation (MT) when compared to other oral potentially malignant disorders (OPMDs) [5], which encompass a variety of lesions associated with a variable risk of MT to invasive cancer. These lesions include PVL, leukoplakia (LE), oral lichen planus (OLP), oral lichenoid lesions, oral erythroplakia, oral submucosal fibrosis, actinic keratosis, palatal lesions in reverse smokers, oral lupus erythematosus (OLE), dyskeratosis songenita (DC), and oral graft versus host disease (OGVHD) [6]. PVL is more prevalent among women [7, 8], typically diagnosed after the age of 60, and is not associated with smoking [7]. The etiology of this condition remains uncertain, although genetic factors and viral infections, such as human papilloma virus, especially types 16 and 18, and Epstein-Barr virus, have been proposed [8]. PVL can progress through multiple stages. Initially, it presents as small, distinct, nonscrapable whitish spots or focal plaques and homogeneous keratotic lesions. Over time, these lesions enlarge, spreading across the mucosal surface. In addition, nonhomogeneous multifocal areas may appear, with a rough surface, featuring exophytic, verrucous, polypoid, or erythematous projections. These changes occur bilaterally, affecting various areas including the buccal mucosa, gum, alveolar ridges, tongue, palatine mucosa, mouth floor, and occasionally the lips [7, 8].

The differential diagnosis for PVL includes frictional keratosis, verrucous hyperplasia, oral squamous cell carcinoma (OSCC), hyperplastic candidiasis, oral hairy leukoplakia, and OLP. In this sense, due to similarities with conventional leukoplakia, PVL cannot definitely recognized based on the histopathologic findings alone, and definitive diagnosis can only be based on high suspicion and temporal, clinical, and histopathologic observations [9].

The aim of this study is to report a case of disseminated PVL, highlighting its clinical-pathological characteristics, disease progression, and to discuss the available diagnostic criteria that make the diagnosis challenging for both the clinician and the pathologist teams.

## 2. Case Report

A black 53-year-old female patient sought dental care at a university center complaining about “white and painful spots in the mouth” and a previous OLP (oral lichen planus) diagnostic, with a 6-month evolution. During anamnesis, the patient reported spontaneous pain and burning sensation within the buccal mucosa, worsening while eating. The patient is diabetic and hypertensive and a smoker for 30 years (10 cigarettes/day). She makes continuous use of metformin 500 mg, glibenclamide 5 mg, losartan 50 mg, and hydrochlorothiazide 25 mg, and the patient is under medical follow-up due to the mentioned systemic conditions.

**Figure 1:**
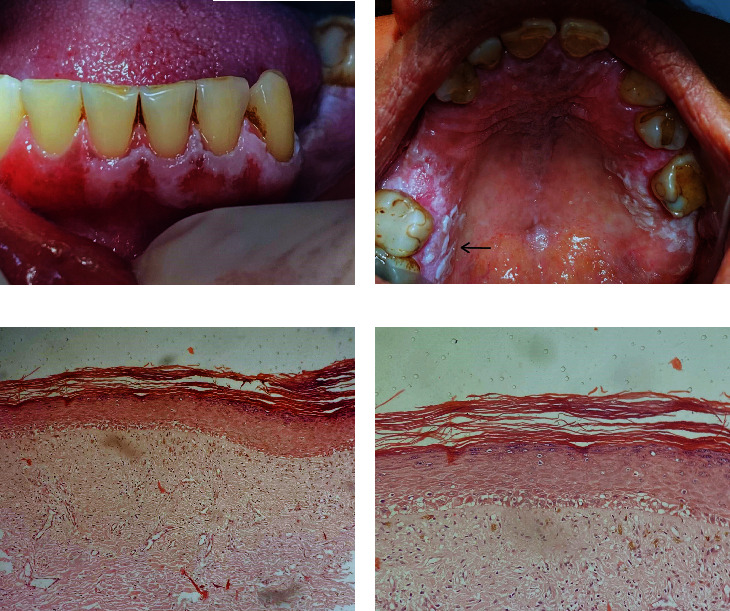
(a) Initial presentation of the patient's oral mucosa: whitish patches enveloping the gingiva. (b) Extensive distribution of white plaques: whitish in hue, they extend seamlessly across the lateral palate, palatine wrinkles, and the alveolar ridge. (c) The epithelium presents atrophy of the epithelial ridges, accompanied by selective thinning of the spinous layer in some regions. A prominent superficial layer of orthokeratin overlays all, alongside disarray within the basal layer. Within the dense underlying connective tissue, pockets of angiogenesis intermingle with a moderate chronic inflammatory infiltrate (H&E—40x). (d) Same area as (c) on higher magnification (H&E—100x).

Whitish, nonscrapable lesions of varying sizes could be seen on intraoral examination, some being circumscribed and others diffuse, distributed over the buccal mucosa, gum (Figure 1(a)), palate, and upper sulcus, close to the upper right molars. In some sites, a verrucous aspect was observed (Figure 1(b)). Based on the patient's symptoms and analysis of the clinical findings, the suggested clinical diagnose was OLP and PVL as differential diagnosis. An incisional biopsy was conducted on the posterior palate (Figure 1(b) (arrow)) for histological analysis. The histological examination revealed a lining epithelium with atrophy of the epithelial ridges, in some areas a decrease in the thickness of the spinous layer and a thick superficial layer of orthokeratin, as well as disorganization of the basal layer. The underlying connective tissue was dense and with areas of angiogenesis and moderate chronic inflammatory infiltrate (Figures 1(c) and 1(d)). The diagnosis report suggested PVL.

**Figure 2:**
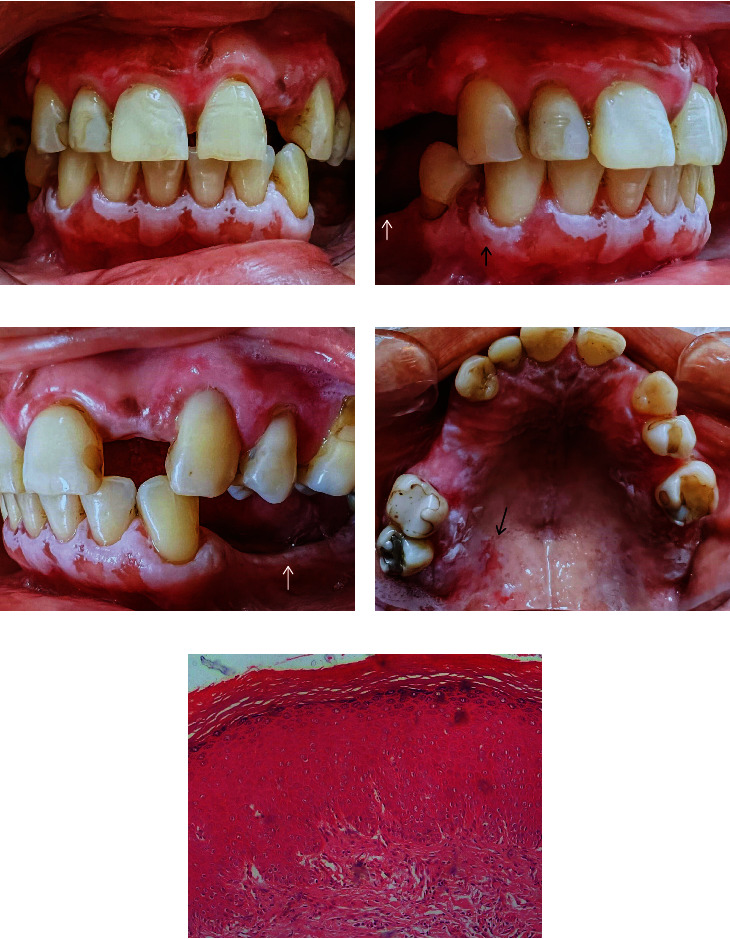
(a) Evolution after a year: the clinical image demonstrates the lesion's thickened state and its encroachment into the gingival tissue surrounding lower teeth. (b) Progressive thickening and expansion: patches in quadrants 3 and 4 display a notable increase in size. (c) Progressive thickening and expansion: lesions in quadrants 2 and 3 display a notable increase in size. (d) Expansive transformations: patches and plaques intensify and thickening, while a diffused erythroplakic region emerges posteriorly on the right side. (e) Microscopic view of alveolar ridge: the mandibular alveolar ridge exhibits bulbous projections towards the connective tissue. This is coupled with stratification loss, an augmented granular layer, and a thick stratum corneum featuring a parakeratin layer. Additionally, basal layer anomalies manifest as pleomorphic cells characterized by abundant cytoplasm and nuclear hyperchromatism (H&E—100x).

During follow-up, the patient received once-daily treatment with triamcinolone acetonide from a dermatology service, yielding unsatisfactory results. After a year, the patient reappeared with increased lesions (Figures 2(a), 2(b) (arrow), and 2(c)) and an erythroplakic area near the prior biopsy site (Figure 2(d) (black arrow)). Although hesitant about another palatal biopsy, the patient underwent 2 incisional biopsies at different mandibular sites: the alveolar ridge and close to the lower left premolars (Figures 2(b) and 2(c) (white arrows)). Anatomopathological examination (Figure 2(e)) showed changes in the lining epithelium characterized by blunt bulbous projections from the connective tissue, loss of stratification, hyperplastic granular layer and stratum corneum with a thick layer of parakeratin and loss of characterization of the basal layer, pleomorphic cells with ample cytoplasm, and nuclear hyperchromatism. The underlying connective tissue was dense, with moderate chronic inflammatory infiltrate. According to the nonspecificity of the histological findings, the diagnosis was suggestive of leukoplakia with mild dysplasia, in both samples.

**Figure 3:**
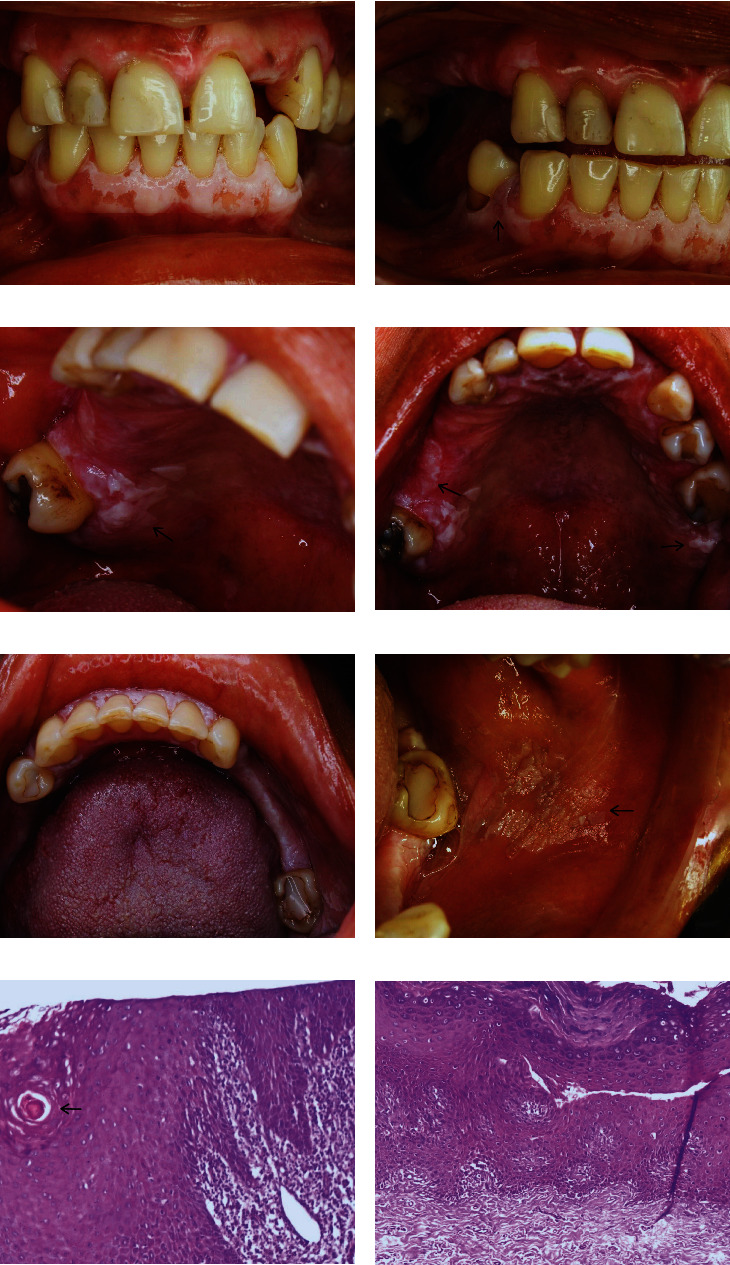
(a) Progression at six months: frontal clinical depiction postulates an amplified area of gingival lesions after six months, following the last incisional biopsies. (b) Lateral evolution: the right lateral view demonstrates lesion progression, serving as a comparative reference to Figures 2(a) and 2(b). (c) Posterior palate clinical display: depiction of the clinical status of the right posterior palate region. (d) Wider clinical perspective: displaying the overall clinical appearances of the palate. (e) Wider clinical perspective: displaying the overall clinical appearances of the mandible. (f) Wider clinical perspective: displaying the overall clinical appearances of the left buccal mucosa. (g) Microscopic detail of S4: histological insight reveals acanthosis, exocytosis, basal layer disorganization, and an intraepithelial keratin pearl (arrow) (H&E—100x). (h) Microscopic observation of S2: highlights include connective tissue projections, acanthosis, hypergranulosis, and a substantial layer of orthokeratin within the histological context (H&E—100x).

After 6 months of the second and third biopsies, due to the gradual increase of the lesions (Figures 3(a), 3(b), 3(c), 3(d), 3(e), and 3(f)) towards previously free areas, the thickening of the oldest lesions and the recurrence in the previously biopsied areas result in the decision to perform new incisional biopsies for follow-up of the evolution, especially regarding the dysplastic areas. Four samples were taken: S1, left jugal mucosa (Figure 3(f)); S2, left alveolar ridge in the maxilla close to the second molar (Figure 3(d)); S3, buccal gingiva between right premolars (Figure 3(b)); and S4, alveolar ridge close to the right third molar (Figure 3(d)). The anatomopathological examination of S4 (Figure 3(g)) showed intense acanthosis, exocytosis, intraepithelial keratinization in the form of a keratin pearl (arrow) and in the form of individual keratinization, a thick layer of orthokeratin on the surface, and disorganization of the basal layer. The underlying connective tissue showed an intense chronic inflammatory infiltration, predominantly composed of lymphocytes, plasmocytes, and histiocytes.

The reports of the remaining samples also demonstrated epithelium displaying blunt projections into the connective tissue, areas of acanthosis, hydropic degeneration, disorganization, and loss of sharpness of the basal layer. In the lamina propria, chronic inflammatory infiltrate predominantly composed of lymphocytes. In the connective tissue, deposition of dense collagen fibers was visualized (Figure 3(h)). The 4 reports were inconclusive due to nonspecific findings. Combining the clinical aspects, the diagnosis was presumptive of PLV.

In summary, the patient underwent a total of 7 biopsies in different areas during three separate surgical procedures over an 18-month follow-up period. Currently, she is under careful monitoring by both a dermatologist and a stomatologist in a specialized hospital setting, while still awaiting scheduling for the removal of dysplastic areas.

## 3. Discussion

In the field of diagnostic protocols for a specific condition, various approaches have been proposed, including those outlined by Hansen et al. [2], Batsakis et al. [10], Cerero-Lapiedra et al. [11], Carrard et al. [12], Villa et al. [13], and Thompson et al. [14], as summarized in Table 1. Despite the abundance of available protocols, our examination reveals inconsistencies in diagnoses, depending on the specific protocol applied. This disparity becomes particularly evident in the present case, where the diagnosis was positive for PVL in two out of the six protocols employed and detailed in this article. The challenge lies in the diverse clinical presentations of the disease and the broad histopathological characteristics that this condition may manifest throughout its progression.

This progression follows somewhat predictable clinicopathological stages. In this regard, Hansen et al. [2] (Table 1) introduced a criteria system consisting of five stages (graded 0-10) that encompass the range of microscopic and clinical appearances. In this system, grade 0 indicates normal oral mucosa. Grade 2 comprises simple hyperkeratosis with minimal or no dysplasia. However, if leukoplakia demonstrates papillary exophytic growth of squamous epithelium, it is categorized as grade 4. In grade 4, no invasion is observed, and the hyperkeratotic epithelium exhibits minimal or no dysplasia. Grade 6 also involves papillary exophytic growth of squamous epithelium, hyperkeratosis, and minimal dysplasia. Additionally, there is a downgrowth of well-differentiated squamous epithelium with broad, blunted rete ridges and intact basement membranes and invasion of the lamina propria. Histologically, a grade 6 lesion cannot be distinguished from verrucous carcinoma. Grade 8 is characterized by exophytic and invasive growth of well-differentiated squamous epithelium with keratin formation and minimal dysplasia. Grade 10 is marked by the loss of cohesion among moderately or poorly differentiated tumor cells, moderate or severe dysplasia is present, and keratin formation is minimal or absent. The infiltrative tumor cells are indistinguishable from a moderately to poorly differentiated squamous cell carcinoma.

Based on the proposed gradations, some samples in our case can be classified as grade 2. This categorization is substantiated by the observation of hyperkeratotic epithelium and clinically evident nonhomogeneous leukoplakia. However, other samples exhibit blunt bulbous projections from the connective tissue and basal layer disorganization, aligning with grade 6 criteria, although not meeting all the specifications for precise grading. Consequently, the histological evaluation based on Hansen et al.'s criteria does not allow for a precise grading of the samples in this case.

Batsakis et al. [10] (Table 1), following the criteria system established by Hansen et al. [2], simplified the histologic stages into five categories, including intermediates. Assessing the current case, according to Batsakis et al.'s [10] criteria, our case is categorized as stage 2. Observing Batsakis et al.'s criteria system, Hansen et al. [2] say that the “controversy in the diagnosis and treatment of verrucous hyperplasia, verrucous carcinoma and squamous cell carcinoma arising is caused by a failure to recognize that, in some cases they are not different and distinct entities, but a pathological process with a continuous spectrum of clinical and histopathological expression” is in agreement with the outcomes observed in our case.

There are several controversies surrounding the diagnosis of PVL. One of them involves differentiating undulating ortho-hyperkeratotic lesions from OLP, which can clinically and histologically resemble PVL. Moreover, epidemiologically, PVL and OLP often manifest in similar populations, particularly women in middle-aged [14], as in this case. These facts can explain the previous misdiagnosis of OLP presented by the patient.

In addition, a noteworthy diagnostic hurdle emerges from the fact that various stages of PVL can coexist within a single specimen or across multiple samples collected simultaneously from the patient [7]. As a result, the diverse histological findings during follow-up and the distinctions between samples obtained concurrently from the patient in this case represent the different stages of transformation through which PVL evolves over time.

Cerero-Lapiedra et al. [11] (Table 1) proposed major and minor criteria to provide a clear and efficient diagnosis of PVL when it is necessary to meet one of the following two conditions: having three main criteria (with E criteria among them) or two main criteria (with E criteria among them) and two minor criteria. In the present case, the patient meets all major criteria and the 2 minor criteria, satisfying and confirming the diagnosis of PVL.

Carrard et al. [12] (Table 1) suggested that simplifying the diagnostic criteria for PVL, proposed by Cerero-Lapiedra et al. [11], eliminates the distinction between major and minor criteria and complies the characteristics into 4 criteria, which must be fully present for a positive diagnosis for PVL.

Given the criteria presented by Carrard et al. [12], the patient fits into the first, second, and fourth criteria. Villa et al. [13] (Table 1) proposed the term “proliferative leukoplakia” to replace the current term and suggested four criteria for the diagnosis of the condition that must be met in full (Table 1). The presented case complies with the four criteria proposed for the diagnosis of PVL since it presents white keratotic and verrucous lesions and multifocal noncontiguous lesions involving many sites, and the lesions progress over time, confirming the PVL diagnosis.

Thompson et al. [14] have developed a guideline that focuses on standardized assessments and reports by pathologists who diagnose PVL-related lesions. The study included 299 biopsies from 84 PVL patients with multifocal oral leukoplakic lesions identified over several years. The guideline recommends the use of standardized histopathologic criteria and descriptive terminology to classify lesions within PVL into four categories. These categories include the following: (1) “corrugated orthohyperkeratotic lesion, not reactive,” (2) “bulky hyperkeratotic epithelial proliferation, not reactive,” (3) “suspicious for,” or “squamous cell carcinoma, and (4) “does not fit any above category.” This case meets the criteria of the second category.

Therefore, in a histopathology level, PVL begins as simple hyperkeratosis, which can progress to verrucous hyperplasia, verrucous carcinoma, and even squamous cell carcinoma. It is important to emphasize that, in cases of multifocality, not all lesions are at the same stage of evolution. While one lesion may be simple hyperkeratosis, another located elsewhere may have already developed into a carcinoma. Therefore, several authors suggest a more rigorous follow-up of these patients, observing possible changes in shape, size, color, and the appearance of new lesions, performing as many biopsies as necessary [11].

According to González-Moles et al. [1], the PVL plaques progressively expand, persistently resisting to treatments and posing a high risk of developing oral cancer. In this context, Bagan et al. [15, 16] highlight the variability of the MT rate, ranging from 40% to 100%, with field cancerization acting as an exacerbating factor. Notably, PVL exhibits a propensity to manifest as oral cancers across distinct sites within the same patient. Among their study participants, 19 individuals with PVL developed oral squamous cell carcinoma (OSCC) during the observation period. Intriguingly, within this subgroup, 52.63% experienced the emergence of at least one additional OSCC, classified as a second primary tumor, over the same period.

In a multicenter study performed by Alabdulaaly et al. [17] which included 86 baseline biopsies from 59 patients, it was found that about one-third of PVL cases did not have oral epithelial dysplasia. In most cases, hyperkeratosis and epithelial atrophy were observed; however, malignant transformation occurred in 3.7% of these sites. Furthermore, it was reported that 47.5% of patients developed carcinoma and the mortality rate was 11.9%. In contrast, Mehanna et al. [18] found that higher grades of dysplasia have significantly higher frequencies of cancer development, and González-Moles et al. [1] observed in their scoping review that epithelial dysplasia appeared in 58% of patients.

Given the high-risk nature of these lesions in terms of malignant progression, the most recent guidelines for managing PVL advocate for regular surveillance, scheduled every 3-6 months. Biopsies should be performed on newly erythroplakic or nodular regions as well as areas of thickened tissue [3]. This approach aligns with the findings of Liu et al. [19], who established a positive correlation between sequential biopsies and different variables such as age, lesion location, lesion type, presence of dysplasia, and the likelihood of malignant transformation. These results underscore the importance of leveraging multiple biopsies and histological examinations to confirm clinical suspicions regarding these high-risk lesions, thus contributing to the timely detection of MT. Consequently, long-term patient follow-up becomes necessary.

It is important to clarify and reflect on certain facts in the Brazilian healthcare scenario. In the context of oral health, dental school clinics play a central role, serving as a crucial hub for promoting health and providing care to the local community, particularly the underprivileged population. These institutions play a fundamental role in supporting the high demands of the Brazilian Unified Health System (SUS). Faced with the inherent complexities of delivering healthcare services in a vast country like Brazil, delays in surgical procedures and the retrieval of examination results within the SUS framework are realities experienced by many patients. In the context of proliferative verrucous leukoplakia (PVL) and other conditions with malignant potential, the speed and effectiveness of diagnosis become imperative to ensure timely treatment and regular monitoring. It is noteworthy that in remote regions such as the Amazon, dental school clinics assume an even more critical role, as the distance from major centers significantly hampers patients' access to specialized medical treatments. Within this context, the current patient, still awaits for the surgical removal of dysplastic areas, underscores the pressing need for effective strategies to expedite diagnoses and treatments, especially in geographically challenging areas, thus ensuring the effectiveness of public health initiatives and the quality of life for patients.

## 4. Conclusion

PVL, an uncommon oral mucosal lesion, carries a significantly elevated risk of progressing into carcinoma. Early detection and continuous monitoring are paramount. Distinguishing PVL from OLP and similar conditions proves challenging due to overlapping clinical signs and histopathological features. Accurate diagnosis and meticulous assessment are vital for atypical oral lesions. Prospective longitudinal studies are essential to refine diagnostic criteria.

## Figures and Tables

**Table 1:** Classification and criteria for oral lesions presented by various authors.

Author (year)	Grades/stage/criteria/category	Conditions	Presented case
Hansen et al. (1985) [2]	Grade 0: normal oral mucosa Grade 2: homogeneous leukoplakia—simple hyperkeratosis with little or no dysplasia. However, when the leukoplakia exhibited papillary exophytic proliferation of squamous epithelium, it was designated grade 4 Grade 4: verrucous hyperplasia—there is no evidence of invasion and the hyperkeratotic epithelium exhibited little or no dysplasia Grade 6: verrucous carcinoma—a papillary exophytic proliferation of squamous epithelium, usually with hyperkeratosis and little or no dysplasia Grade 8: papillary squamous carcinoma—characterized by an exophytic and invasive growth of well-differentiated squamous epithelium with keratin formation and minimal dysplasia Grade 10: less differentiated carcinoma—loss of cohesion of moderately differentiated or poorly differentiated tumor cells. There was moderate or severe dysplasia-differentiated tumor cells; moderate or severe dysplasia and keratin formation was minimal or absent; tumor cells were infiltrative and indistinguishable from a moderately differentiated to poorly differentiated squamous cell carcinoma		Grade 2 (for some samples)
Batsakis et al. (1999) [10]	Stage 1: clinical flat leukoplakia without dysplasia Stage 2: nonhomogenous leukoplakia with dysplasia Stage 3: verrucous hiperplasia Stage 4: verrucous carcinoma Stage 5: conventional squamous cell carcinoma		Stage 2
Cerero-Lapiedra et al. (2010) [11]	Major criteria (MC): (A) Leukoplakia lesion in more than two different oral sites, which is most common in the gingiva, alveolar ridges, and palate (B) Existence of a verrucous area (C) Lesions have spread or thickened during the disease (D) Recurrence in a previously treated area (E) Presence of simple epithelial hyperkeratosis, verrucous hyperplasia, verrucous carcinoma, or OSCC, either in situ or infiltrating, at the histopathological level	(1) Three major criteria (being E among them) or (2) Two major criteria (being E among them) + two minor criteria	(A) Meets the criteria (B) Meets the criteria (C) Meets the criteria (D) Meets the criteria (E) Meets the criteria
	Minor criteria (MC): (A) An oral leukoplakia lesion that occupies at least 3 cm when putting together all the affected areas (B) That the patient be female (C) That the patient (male or female) be a nonsmoker (D) A disease evolution superior to 5 years		(A) Meets the criteria (B) Meets the criteria (C) Does not meet the criteria (D) Does not meet the criteria Total: 5 major criteria including “E” and 2 minor criteria
Carrard et al. (2013) [12]	(1) Leukoplakia showing the presence of verrucous or wartlike areas, involving more than two oral subsites. The following oral subsites are recognized: dorsum of the tongue (unilateral or bilateral), border of the tongue, cheek mucosa, alveolar mucosa or gingiva upper jaw, and alveolar mucosa or gingiva lower jaw. Hard and soft palate, floor of the mouth, upper lip, and lower lip (2) When adding all involved sites, the minimum seize should be at least three centimeters (3) A well-documented period of disease evolution of at least five years, being characterized by spreading and enlarging and the occurrence of one or more recurrences in a previously treated area (4) The availability of at least one biopsy to rule out the presence of a verrucous carcinoma or squamous cell carcinoma	The 4 criteria must be fully present for a positive diagnosis for PVL	(1) Meets the criteria (2) Meets the criteria (3) Partially meets the criteria (4) Meets the criteria
Villa et al. (2018) [13]	(1) White keratotic lesions that may be smooth, fissured, verrucous, or erythematous with or without ulcer (2) Multifocal noncontiguous lesions OR a single large lesion > 4.0 cm involving one site OR a single large lesion>3 cm involving contiguous sites (3) Lesions that progress/expand in size and/or develop multifocality over time (4) Histopathology that if not overtly exhibiting dysplasia or carcinoma shows hyperkeratosis, parakeratosis, atrophy, or acanthosis with minimal to no cytologic atypia, with or without a lymphocytic band, OR verrucous hyperplasia; these features must not support a diagnosis of frictional or reactive keratosis		(1) Meets the criteria (2) Meets the criteria (3) Meets the criteria (4) Meets the criteria
Thompson et al. (2021) [14]	(1) Corrugated orthohyperkeratotic lesion, not reactive (2) Bulky hyperkeratotic epithelial proliferation, not reactive (3) “Suspicious for,” or “squamous cell carcinoma” (4) Does not fit any above category		2° category

## Data Availability

The data from this study are fully available in this article.
